# A dataset of 40 assembled and annotated transcriptomes from 34 species in *Silene* and related genera

**DOI:** 10.1016/j.dib.2024.111094

**Published:** 2024-11-01

**Authors:** Patrik Cangren, Yann J.K. Bertrand, John M. Braverman, Gregor Duncan Gilfillan, Matthew B. Hamilton, Bengt Oxelman

**Affiliations:** a Department of Biological and Environmental Sciences, University of Gothenburg, Medicinaregatan 7B, Goteborg 413 90, Sweden; b Laboratory of Molecular Biology and Bioinformatics. Institute of Botany, Academy of Sciences of the Czech Republic, CZ-252 43 Průhonice, Czech Republic; c Department of Biology, The Science Center, Saint Josephs University, 5600 City Ave. Philadelphia, PA 19131, USA; d Department of Medical Genetics, Oslo University Hospital and University of Oslo, Kirkeveien 166, 0450 Oslo, Norway; e Department of Biology, Georgetown University, 37th and O Streets NW Washington, DC 20057, USA

**Keywords:** Genomics, Phylogenetics, RNA-transcripts, Nucleotide, Assembly, Functional-annotation, Sileneae

## Abstract

A dataset of 40 assembled and annotated transcriptomes from 34 different species sampled from phylogenetically diverse parts of the flowering plant genus *Silene* (Caryophyllaceae) and the related genera *Agrostemma, Atocion, Eudianthe, Heliosperma, Petrocoptis* and *Viscaria.* RNA extracted from roots, stems, leaves, buds and flowers were sequenced using paired end reads on the Illumina Hiseq platform. A total of 716 million raw reads were produced and assembled into 2.67 million isogroups (“genes”). Contigs from all samples were annotated using UniProt/SwissProt and assigned with GO-terms. A total of 974274 annotations were made (per sample average 24357, stdev 7034), giving an annotation proportion of 37% (per sample average 39%, stdev 9.75%). 741087 of the annotations had taxonomic identities within Magnoliopsida (per sample average 18527, stdev 3931), resulting in assignment of 4519488 GO-terms (per sample average 112987, stdev 22536). The data set can be further utilized for biological research and phylogenetic studies, evolutionary questions, functional analyses of genes, polyploidy as well as for marker development.

Specifications Table

**Table:** 

Subject	Biological Sciences
Specific subject area	Phylogeny and Evolution
Type of data	Raw sequence data, Processed sequence data, Annotation information, Filtered Annotations, Tables
Data collection	Samples were grown from seeds in the botanical gardens in Gothenburg and Copenhagen or collected in the wild and preserved with RNAlater (ThermoFisher). Sequencing libraries were constructed from RNA extracted from all major parts of the plants and sequenced on the Illumina HiSeq platform. Adapter removal and quality filtering of raw reads were performed using Trimmomatic. Filtered reads were assembled with Trinity v.2.9.1 using default settings. Assembled contigs were searched against UniProt/Swiss-Prot using BLASTx v. 2.2.31+. Annotated contigs were filtered to exclude hits for accessions with taxa outside of Angiosperms. Information on assigned GO-terms for each filtered hit were downloaded from UniProt/Swiss-Prot.
Data source location	Department of Biological and Environmental Sciences, University of Gothenburg, Sweden
Data accessibility	Repository name: Mendeley Data Data identification number: 10.17632/vykf3g4z5g.2 Direct URL to data: https://data.mendeley.com/datasets/vykf3g4z5g/2
Related research article	None*.*

## 1 Value of the Data

• These data contain the largest collection of transcriptomes from the genus *Silene* published to date and include a phylogenetically diverse sample of species.
• The genus *Silene* is widely used as a model to study a variety of both ecological and evolutionary questions such as breeding systems, population genetics, invasiveness, heavy metal tolerance, speciation, sex determination, evolution of sex chromosomes and organelle evolution.
• Additional transcriptomes from the related genera *Agrostemma, Atocion, Eudianthe, Heliosperma, Petrocoptis* and *Viscaria* within *Sileneae* allow for further phylogenetic studies on the relationships and marker development within the larger taxonomic group.
• Researchers can utilize the data to study a diverse range of biological and evolutionary questions such as phylogenetics, polyploidy, functional roles of genes and genome evolution. It can also be used for primer and marker development.

## 2 Background

The data set provided here were initially generated as a joint effort between researchers from different fields. There were multiple goals: i. to include species from various sections across the phylogenetic diversity in order to fill taxonomic gaps between previously sequenced transcriptomes, ii. the creation of a large-scale genomic resource to locate single-copy genes, iii. facilitate development of a marker set for sequence capture to enable further collection of data, iv. phylogenetic inference, v. studying variation in substitution rates between annual and perennial taxa and vi. the use of polyploid taxa to explore the evolution of genomes after polyploidization events. The data has previously been utilized for phylogenetic studies, creation of a *Silene* specific set of 48 probes for sequence capture and an initiative to create a new data-base portal for genomic resources in *Silene.* The sampling includes 27 species from *Silene* of which 24 are diploid and three are polyploid, together with seven species from the related genera *Agrostemma, Atocion, Eudianthe, Heliosperma, Petrocoptis and Viscaria.* The dataset contains raw sequencing reads as well as assembled and annotated contigs for all included samples.

## 3 Data Description

The data presented in this article are divided into 2 folders: one for assembled contigs and one for annotation information. Four additional tables are included with information on included samples, sequencing results, assembled contigs and annotations. Raw sequencing reads for all included samples are available as a BioProject at the NCBI Sequence Read Archive (SRA) under accession number PRJNA1124948 (https://www.ncbi.nlm.nih.gov/sra/PRJNA1124948).

### 3.1 Sampling

Table 1 shows information on included samples taxon, locality, sample ID, input material and SRA-accession for the raw sequencing reads of each sample. The table is organized according to the classification of Jafari et al. [1].

**Table 1:** Included samples with species names and taxonomic affiliation according to Jafari et al. (2020), country of origin, collection information, sample ID, input material type and SRA accession ID for raw sequencing read data.

*Genus, subgenus, section, species*	Locality	Additional collection Info	Sample ID	Input material	SRA Accession
***Agrostemma* L.**					
*Agrostemma githago* L.	Hisingen, Sweden		P451_124	RNAlater	SAMN41876039
***Atocion* Adans.**					
*Atocion armeria* (L.) Raf.	Vila Cha, Portugal	Cultivated in the Gothenburg Botanical Garden, from seeds collected in the field.	P451_102	Frozen	SAMN41876039
*Atocion rupestre* (L.) Oxelman	Bohuslän, Hallinden, Sweden		P451_121	Fresh	SAMN41876041
***Eudianthe* (Rchb.) Rchb.**					
*Eudianthe laeta* Rchb. ex Willk.	Denmark	Cultivated in the Copenhagen Botanical Garden	P451_128	Frozen	SAMN41876042
***Heliosperma* (Rchb.) Rchb.**					
*Heliosperma macranthum* Pančić	Komovi, Montenegro	Cultivated in the Gothenburg Botanical Garden, from seeds collected in the field.	P451_115	Fresh	SAMN41876043
***Petrocoptis* A. Braun ex Endl.**					
*Petrocoptis crassifolia* Rouy	Burrow - Central Pyrenees, Spain	Cultivated in the Gothenburg Botanical Garden from seeds collected in the field	P451_126	Frozen	SAMN41876044
***Viscaria* Bernh.**					
*Viscaria vulgaris* Bernh.	Bohuslän, Sotenäs, Sweden		P1844_106	Fresh	SAMN41876045
***Silene* L.**					
Incertae sedis					
*S.* sect. *Atocion* Otth.					
*Silene assyriaca* Hausskn. & Bornm. ex Lazkov	Akarsu Village, Turkey	Cultivated in the Gothenburg Botanical Garden from seeds collected in the field	P451_103	Fresh	SAMN41876046
*Silene atocioides* Boiss.	Antalya-Altinyaka, Turkey	Cultivated in the Gothenburg Botanical Garden from seeds collected in the field	P451_104	Fresh	SAMN41876047
*Silene fraudatrix* Meikle	Lefkosa, Alevkayasi, Halevga, Cyprus	Cultivated in the Gothenburg Botanical Garden from seeds collected in the field	P451_113	Fresh	SAMN41876048
Subgenus Lychnis					
*S.* sect *Coccyganthe*					
*Silene flos-cuculi* L.	Bohuslän, Sotenäs, Sweden		P451_112	Fresh	SAMN41876049
Subgenus*Behenantha*(Otth) Torr. & A. Gray					
*S.* sect. *Behenantha* Otth.					
*Silene behen* L.	Mersin, Turkey	Cultivated in the Gothenburg Botanical Garden from seeds collected in the field	P451_127	Frozen	SAMN41876050
*S.* sect. *Conoimorpha* Otth. in Candolle					
*Silene conoidea* L.	Vazrab river, Lalon, Tehran, Iran	Cultivated in the Gothenburg Botanical Garden from seeds collected in the field	P1844_105	Fresh	SAMN41876051
*Silene conoidea* L.	Vazrab river, Lalon, Tehran, Iran	Cultivated in the Gothenburg Botanical Garden from seeds collected in the field	P451_107F	Fresh	SAMN41876052
*S.* sect. *Cryptoneurae* Aydin & Oxelman					
*Silene ertekinii* Aydin & Oxelman	Antalya to Altinyaka, Turkey	Cultivated in the Gothenburg Botanical Garden from seeds collected in the field	P451_109	Fresh	SAMN41876053
*Silene ertekinii* Aydin & Oxelman	Antalya to Altinyaka, Turkey	Cultivated in the Gothenburg Botanical Garden from seeds collected in the field	P451_110	Fresh	SAMN41876054
*S.* sect. *Dichotomae* (Rohrb.) Chowdhuri					
*Silene dichotoma* Ehrh.	Sutlegen, Turkey	Cultivated in the Gothenburg Botanical Garden from seeds collected in the field	P451_135	Frozen	SAMN41876055
*S.* sect. *Elisanthe* (Fenzl ex Endl.) Ledeb.					
*Silene noctiflora* L.	Bohuslän, Hovenäset, Stavsäng, Sweden		P451_117	Fresh	SAMN41876056
*S.* sect. *Sedoides* Oxelman & Greuter					
*Silene sedoides* Poir.	Plimiri, Rodos, Greece.	Cultivated in the Gothenburg Botanical Garden from seeds collected in the field	P451_122	Fresh	SAMN41876057
*S.* sect. *Odontopetalae* Chowdhuri					
*Silene odontopetala* Fenzl	Tuchal mountain, Tehran, Iran	Cultivated from seeds collected in the field	P451_119	Fresh	SAMN41876058
*S.* sect. *Physolychnis* (Benth.) Bocquet in Candollea					
*Silene ajanensis* (Regel & Tiling) Vorosch	Batarejanaja, Russia		BOX5120	RNAlater	SAMN41876059
*Silene involucrata* (Cham. & Schltdl.) Bocquet *subsp. furcata* (Raf.) V.V.Petrovsky & Elven	Greenland		BOX4906	Frozen	SAMN41876060
*Silene involucrata* (Cham. & Schltdl.) Bocquet *subsp. furcata* (Raf.) V.V.Petrovsky & Elven	Spitsbergen. Adventdalen, Endalen, Norway		BOX4907	Frozen	SAMN41876061
*Silene involucrata* (Cham. & Schltdl.) Bocquet *subsp. tenella* (Tolmatchew) Bocquet	Abisko, Sweden		BOX4908	Frozen	SAMN41876062
*Silene involucrata* (Cham. & Schltdl.) Bocquet	“Ary-Mas” nature reserve, Severo-Sibirskaya Nizmennost, Russia	Cultivated from seeds	BOX4909	RNAlater	SAMN41876063
*Silene involucrata* (Cham. & Schltdl.) Bocquet subsp. involucrata	“Ary-Mas” nature reserve, Severo-Sibirskaya Nizmennost, Russia	Cultivated from seeds	BOX5115	RNAlater	SAMN41876064
*Silene linneana* Voroschilov	Kil'demtsy village,Sakha republic, Yakutsk, Russia		BOX4911	Frozen	SAMN41876065
*Silene sachalinensis* F. Schmidt	?	Cultivated from seeds labelled S. araratica from Zdeneks garden	BOX5117	RNAlater	SAMN41876066
*Silene soczaviana* Schischk.	Mulgrave hills, Canada		BOX4885	Frozen	SAMN41876067
Subgenus*Silene*					
*S.* sect*. Arenosae* Eggens, F.Jafari & Oxelman					
*Silene exsudans* Boiss. & Heldr.	Kizilot, Antalya Turkey	Cultivated in Gothenburg Botanical Garden from seeds collected in the field	P451_129	Frozen	SAMN41876068
*S.* sect. *Auriculatae* (Boiss.) Schischk. in Komarov					
*Silene commelinifolia* Boiss.	Tuchal Mountain, Tehran, Iran	Cultivated from seeds collected in the field	P451_106	Fresh	SAMN41876069
*Silene eriocalycina* Boiss.	Lahderaz, Sabz kouh, Morchegen, Gandoman, Iran	Cultivated in Gothenburg Botanical Garden from seeds collected in the field	P451_108F	Fresh	SAMN41876070
*S.* sect. *Muscipula* (Tzvelev) Oxelman, F.Jafari & Gholipour					
*Silene muscipula* L.	Denmark	Cultivated in Copenhagen Botanical Garden	P451_136	Frozen	SAMN41876071
*S.* sect. *Sclerocalycinae* (Chowdhuri) F.Jafari, Oxelman & Rabeler					
*Silene laxa* Boiss. & Kotschy	Firouzkouh, Tehran, Iran	Cultivated in Gothenburg Botanical Garden from seed collected in the field	P451_114	Fresh	SAMN41876072
*Silene vittata* Stapf	Pass between Isamlar and Ikizçe, Turkey	Cultivated in Gothenburg Botanical Garden from seeds collected in the field	P451_123	Fresh	SAMN41876073
*S.* sect. *Silene*					
*Silene ciliata* Pourr.	Bengt Oxelman 2631 (GB), Origin unknown.	Cultivated in Uppsala Botanical Garden from seeds obtained from Paris.	P451_101	Fresh	SAMN41876074
*Silene colorata* Poir.	Gaziantep, Turkey	Cultivated in Gothenburg Botanical Garden from seed collected in the field	P451_105	Unknown	SAMN41876075
*S.* sect. *Siphonomorpha* Otth.					
*Silene acaulis* (L.) L.	Mt Torkilstöten, Sweden	Cultivated at Gothenburg Botanical Garden from seeds collected in the field	P451_125	Frozen	SAMN41876076
*Silene nutans* L.	Bohuslän, Hovenäset, Stavsäng, Sweden		P451_118	Fresh	SAMN41876077
*S.* sect. *Rigidulae* (Boiss.) Schischk. in Komarov					
*Silene echinospermoides* Hub.-Mor.	North of Marmaris, Turkey	Cultivated at Gothenburg Botanical Garden from seeds collected in the field	P451_111	Frozen	SAMN41876078

### 3.2 Sequencing

The dataset contained an average of 17.47 million (stdev 6.46 million) raw read pairs per sample. After adapter removal and quality filtering an average of 15.5 million (stdev 5.63 million) read pairs remained per sample. In total the dataset contains 716 million raw read pairs (1.68E+15 bases) with 638 million read pairs (1.45E+11 bases) left after filtering and trimming. In addition to the paired reads a total of 62.2 million single reads passed the quality filtering, of which 51.2 million were forward and 11.0 million reverse. The average read length after trimming was 92.66 bp (stdev 3.19). Full information per sample is presented in Table 2.

**Table 2:** Quality filtering. Number of input read pairs, output read pairs, output single reads forward / reverse and completely dropped read pairs.

*Species name*	Sample ID	Input read pairs	Output read pairs	Output single reads forward	Output single reads reverse	Dropped read pairs
*Agrostemma githago* L.	P451_124	24112942	22725633	971990	228283	187036
*Atocion armeria* (L.) Raf.	P451_102	11315337	10326278	720105	95624	173330
*Atocion rupestre* (L.) Oxelman	P451_121	22294313	20787517	1123477	197484	185835
*Eudianthe laeta* Rchb. ex Willk.	P451_128	19625259	18518825	763093	184848	158493
*Heliosperma macranthum* Pančić	P451_115	14340190	12906795	1128388	133252	171755
*Petrocoptis crassifolia* Rouy	P451_126	21398313	20034609	982066	194556	187082
*Silene acaulis* (L.) L.	P451_125	18945308	17665870	943253	169017	167168
*Silene ajanensis* (Regel & Tiling) Vorosch	BOX5120	16377073	14191350	1143281	304424	738018
*Silene assyriaca* Hausskn. & Bornm. ex Lazkov	P451_103	9127467	8017710	888069	95346	126342
*Silene atocioides* Boiss.	P451_104	11545292	10134467	1089149	118532	203144
*Silene behen* L.	P451_127	20388695	19140048	912968	159650	176029
*Silene ciliata* Pourr.	P451_101	10192980	9072528	905500	92001	122951
*Silene colorata* Poir.	P451_105	11551324	10181680	1082283	118589	168772
*Silene commelinifolia* Boiss.	P451_106	11462296	10323693	898319	109373	130911
*Silene conoidea* L.	P1844_105	39775814	32271499	5846279	750065	907971
*Silene conoidea* L.	P451_107F	15780766	13930324	1421028	162271	267143
*Silene dichotoma* Ehrh.	P451_135	17638547	16439052	891646	153865	153984
*Silene echinospermoides* Hub.-Mor.	P451_111	10876570	9831870	829705	83976	131019
*Silene eriocalycina* Boiss.	P451_108F	13027577	11633227	1102455	113064	178831
*Silene ertekinii* Aydin & Oxelman	P451_109	13564252	12070759	1170484	141247	181762
*Silene ertekinii* Aydin & Oxelman	P451_110	12400320	11192693	968714	107496	131417
*Silene exsudans* Boiss. & Heldr.	P451_129	17433016	16240847	835407	192966	163796
*Silene flos-cuculi* L.	P451_112	14825777	13247534	1190136	173151	214956
*Silene fraudatrix* Meikle	P451_113	14586134	12674162	1522763	179032	210177
*Silene involucrata* (Cham. & Schltdl.) Bocquet	BOX4909	15772276	13868507	730994	789550	383225
*Silene involucrata* (Cham. & Schltdl.) Bocquet *subsp. furcata* (Raf.) V.V.Petrovsky & Elven	BOX4906	17691017	15626558	1296233	326189	442037
*Silene involucrata* (Cham. & Schltdl.) Bocquet subsp. involucrata	BOX5115	16174005	13912120	1115081	284340	862464
*Silene involucrata* (Cham. & Schltdl.) Bocquet *subsp. furcata* (Raf.) V.V.Petrovsky & Elven	BOX4907	15515562	13447355	840011	798114	430082
*Silene involucrata* (Cham. & Schltdl.) Bocquet *subsp. tenella* (Tolmatchew) Bocquet	BOX4908	16387696	13870928	1174916	280392	1061460
*Silene laxa* Boiss. & Kotschy	P451_114	11985993	10666366	986347	156048	177232
*Silene linneana* Voroschilov	BOX4911	14998150	13181484	689295	776694	350677
*Silene muscipula* L.	P451_136	32821341	30821716	1396750	356917	245958
*Silene noctiflora* L.	P451_117	15592092	14084073	1201144	133331	173544
*Silene nutans* L.	P451_118	13748725	12302911	1149086	126603	170125
*Silene odontopetala* Fenzl	P451_119	25678045	23663427	1481241	285179	248198
*Silene sachalinensis* F. Schmidt	BOX5117	17844665	12638566	1273424	280157	3652518
*Silene sedoides* Poir.	P451_122	19797649	18430258	998932	179618	188841
*Silene soczaviana* Schischk.	BOX4885	14069842	12067058	662427	712226	628131
*Silene vittata* Stapf	P451_123	18034049	16740902	950112	162301	180734
*Viscaria vulgaris* Bernh.	P1844_106	34880003	28208434	4869669	915263	886637

Table 2 shows information on sequencing results before and after trimming and quality filtering.

### 3.3 Assembled sequence data

Assembled sequence data is organized into separate subfolders for each sample. Within each sample folder two fasta files are provided. The original trinity output fasta which contains all isoforms assembled by Trinity and one with the suffix “_LongestIsoform” which includes only the longest contig from each isogroup. The accession lines of all fasta files consists of the sample id followed by the unedited trinity accession, separated by an underscore.

### 3.4 Assembly

RNA-seq assembly using trinity assembled an average of 81.9 million bases per sample. The number of isogroups varied between 1852 and 112989 with an average of 66512 (stdev 24479) and the number of isoforms per sample varied between 2950 and 185263 with an average of 95697(stdev 40233). In total 17.1% of the isogroups contained more than one isoform and for individual samples the percentage varied between 6.6% and 25.4% with an average of 16.3.%. The average contig length for individual samples varied between 447 and 1093 bases, with a total average length for the entire dataset of 812 bases. The maximum transcript length varied between 6123 and 66970 bases with an average max length of 25407(stdev 12026). In total the full dataset assembled 3.28 E+9 bases and yielded a total of 2.67 million isogroups (“genes”) and 3.83 million isoforms (transcripts) with a minimum length of 100bp. Full information on assembly statistics is presented in Table 3.

**Table 3:** Assembly statistics. Number of transcripts (Trinity “Genes” and transcripts), N50, Median and average contig lengths, total assembled bases.

*Species name*	Sample ID	Isogroups	Isoforms	N50	Median Contig Length	Average Contig Length	Total Assembled Bases
*Agrostemma githago* L.	P451_124	112989	185263	1498	472	868	160719834
*Atocion armeria* (L.) Raf.	P451_102	58474	79449	1595	515	930	73877413
*Atocion rupestre* (L.) Oxelman	P451_121	82529	117865	1685	504	952	112235427
*Eudianthe laeta* Rchb. ex Willk.	P451_128	67976	91847	1490	460	860	79022445
*Heliosperma macranthum* Pančić	P451_115	65136	80821	1124	413	723	58432249
*Petrocoptis crassifolia* Rouy	P451_126	88757	122006	1666	492	939	114528358
*Silene acaulis* (L.) L.	P451_125	91333	128327	1517	470	876	112471120
*Silene ajanensis* (Regel & Tiling) Vorosch	BOX5120	87421	130694	1442	476	852	111361553
*Silene assyriaca* Hausskn. & Bornm. ex Lazkov	P451_103	41395	49193	782	387	601	29581683
*Silene atocioides* Boiss.	P451_104	36083	45276	715	387	568	25709072
*Silene behen* L.	P451_127	70524	90851	1487	465	866	78665191
*Silene ciliata* Pourr.	P451_101	70986	99263	1035	395	682	67703909
*Silene colorata* Poir.	P451_105	31586	36834	755	384	581	21410352
*Silene commelinifolia* Boiss.	P451_106	57043	77099	1214	436	764	58901346
*Silene conoidea* L.	P1844_105	88259	120272	1436	435	804	96709979
*Silene conoidea* L.	P451_107F	26144	29417	779	376	591	17372331
*Silene dichotoma* Ehrh.	P451_135	89354	130097	1688	504	952	123882704
*Silene echinospermoides* Hub.-Mor.	P451_111	54081	71322	1473	478	864	61611743
*Silene eriocalycina* Boiss.	P451_108F	77185	109611	1330	453	807	88457241
*Silene ertekinii* Aydin & Oxelman	P451_109	22451	26257	776	386	594	15596750
*Silene ertekinii* Aydin & Oxelman	P451_110	58149	78853	1477	477	873	68819362
*Silene exsudans* Boiss. & Heldr.	P451_129	67199	90683	1434	449	839	76097664
*Silene flos-cuculi* L.	P451_112	32538	37338	997	425	683	25487348
*Silene fraudatrix* Meikle	P451_113	18657	21779	720	396	573	12480521
*Silene involucrata* (Cham. & Schltdl.) Bocquet	BOX4909	77296	131405	1547	572	946	124244316
*Silene involucrata* (Cham. & Schltdl.) Bocquet *subsp. furcata* (Raf.) V.V.Petrovsky & Elven	BOX4906	92092	151223	1466	522	884	133636038
*Silene involucrata* (Cham. & Schltdl.) Bocquet subsp. Involucrate	BOX5115	72828	104128	1537	532	914	95209265
*Silene involucrata* (Cham. & Schltdl.) Bocquet *subsp. furcata* (Raf.) V.V.Petrovsky & Elven	BOX4907	91084	135930	1291	480	801	108892924
*Silene involucrata* (Cham. & Schltdl.) Bocquet *subsp. tenella* (Tolmatchew) Bocquet	BOX4908	80289	133903	1546	556	935	125231716
*Silene laxa* Boiss. & Kotschy	P451_114	1852	2950	470	319	447	1319380
*Silene linneana* Voroschilov	BOX4911	59027	90641	1707	623	1026	92973979
*Silene muscipula* L.	P451_136	90084	131884	1640	494	931	122787311
*Silene noctiflora* L.	P451_117	76525	107732	1471	477	867	93391757
*Silene nutans* L.	P451_118	64147	88850	1365	435	802	71251413
*Silene odontopetala* Fenzl	P451_119	102476	138975	1314	406	761	105790925
*Silene sachalinensis* F. Schmidt	BOX5117	74045	121013	1421	535	880	106471762
*Silene sedoides* Poir.	P451_122	78619	123180	1873	623	1093	134679534
*Silene soczaviana* Schischk.	BOX4885	72234	117803	1440	529	880	103674865
*Silene vittata* Stapf	P451_123	65215	88901	1282	463	803	71379877
*Viscaria vulgaris* Bernh.	P1844_106	73776	108957	1425	542	872	94985456

Table 3 shows detailed assembly results with number of isogroups/isoforms, N50, median and average contig length and total assembled bases.

### 3.5 Annotation data

Annotation information is organized into separate subfolders for each sample. For each sample three files are provided: the raw BLASTx output in .xml format and two tab separated text files, one with all first hits accessions and one which includes only the contigs with hits against accessions within Angiosperms. The files are designed to support easy format conversion or loading into sql databases.

The tab separated text data files contain information in the following order and format: Contig ID (Sample ID followed by Contig Name), Sample ID, Contig Name, Contig Length, Contig Sequence, BLAST Hit Accession, BLAST Hit Definition, BLAST Hit Length, BLAST Hit Sequence, BLAST Hit GO-Terms, BLAST Hit Organism, BLAST Hit Taxon X-Ref, BLAST Hit Organism Classification. The files contain headers describing the columns.

### 3.6 Annotation

BLASTx search against UnitProt/Swiss-Prot resulted in a total of 974274 hits where 741087 had a taxonomic classification within Magnoliopsida with 719700 of these being annotated with GO-terms. Annotations were distributed across a total of 65026 unique accessions of which 21738 belonged to organisms with taxonomic classification within Magnoliopsida. On average 39% of contigs were annotated for each sample, with 78% of the annotations having a taxonomic identity within Magnoliopsida resulting in assignment of GO-terms to 76% of the annotated contigs. The most common taxon hit during BLAST searching with these contigs was *Arabidopsis thaliana* with 579826 hits, followed by *Oryza sativa* supsp. japonica at 35744 hits and *Homo sapiens* 20363 hits.

Table 4 shows detailed annotation results for each sample with number of contigs with BLAST hits in UniProt/Swiss-Prot, number of hits with taxonomic identity within Magnoliopsida and number of contigs with GO-terms assigned.

**Table 4:** Annotation results: Total contigs, Contigs w. Blast hits, Contigs with hits within Magnoliopsida, Contigs annotated with GO-Terms.

*Species name*	Sample ID	Contigs	Total hits	% hits	Hits within Magnoliopsida	% of hits within Magnoliopsida	Hits within Magnoliopsida annotated w. GO-terms.	% of hits annotated with GO-terms
*Agrostemma githago* L.	P451_124	112989	32988	29,2	20866	63,3	20287	61,5
*Atocion armeria* (L.) Raf.	P451_102	58474	19867	34,0	17620	88,7	17095	86,0
*Atocion rupestre* (L.) Oxelman	P451_121	82529	22919	27,8	19323	84,3	18769	81,9
*Eudianthe laeta* Rchb. ex Willk.	P451_128	67976	23028	33,9	17712	76,9	17235	74,8
*Heliosperma macranthum* Pančić	P451_115	65136	21571	33,1	18761	87,0	18219	84,5
*Petrocoptis crassifolia* Rouy	P451_126	88757	28312	31,9	18450	65,2	17956	63,4
*Silene acaulis* (L.) L.	P451_125	91333	28269	31,0	20578	72,8	19894	70,4
*Silene ajanensis* (Regel & Tiling) Vorosch	BOX5120	87421	34298	39,2	19899	58,0	19306	56,3
*Silene assyriaca* Hausskn. & Bornm. ex Lazkov	P451_103	41395	22079	53,3	19076	86,4	18636	84,4
*Silene atocioides* Boiss.	P451_104	36083	18787	52,1	15639	83,2	15307	81,5
*Silene behen* L.	P451_127	70524	26537	37,6	18152	68,4	17632	66,4
*Silene ciliata* Pourr.	P451_101	70986	26838	37,8	20113	74,9	19500	72,7
*Silene colorata* Poir.	P451_105	31586	17927	56,8	15247	85,1	14999	83,7
*Silene commelinifolia* Boiss.	P451_106	57043	22018	38,6	19018	86,4	18427	83,7
*Silene conoidea* L.	P1844_105	88259	38833	44,0	21197	54,6	20668	53,2
*Silene conoidea* L.	P451_107F	26144	16057	61,4	12994	80,9	12770	79,5
*Silene dichotoma* Ehrh.	P451_135	89354	28533	31,9	19900	69,7	19180	67,2
*Silene echinospermoides* Hub.-Mor.	P451_111	54081	21180	39,2	18128	85,6	17561	82,9
*Silene eriocalycina* Boiss.	P451_108F	77185	24085	31,2	20069	83,3	19388	80,5
*Silene ertekinii* Aydin & Oxelman	P451_109	22451	13538	60,3	12208	90,2	11956	88,3
*Silene ertekinii* Aydin & Oxelman	P451_110	58149	21005	36,1	18573	88,4	18005	85,7
*Silene exsudans* Boiss. & Heldr.	P451_129	67199	21650	32,2	17875	82,6	17437	80,5
*Silene flos-cuculi* L.	P451_112	32538	18865	58,0	16826	89,2	16487	87,4
*Silene fraudatrix* Meikle	P451_113	18657	12072	64,7	10925	90,5	10733	88,9
*Silene involucrata* (Cham. & Schltdl.) Bocquet	BOX4909	77296	25367	32,8	22540	88,9	21817	86,0
*Silene involucrata* (Cham. & Schltdl.) Bocquet *subsp. furcata* (Raf.) V.V.Petrovsky & Elven	BOX4906	92092	31445	34,1	23750	75,5	23014	73,2
*Silene involucrata* (Cham. & Schltdl.) Bocquet subsp. involucrata	BOX5115	72828	29968	41,1	19534	65,2	18896	63,1
*Silene involucrata* (Cham. & Schltdl.) Bocquet *subsp. furcata* (Raf.) V.V.Petrovsky & Elven	BOX4907	91084	39572	43,4	22355	56,5	21668	54,8
*Silene involucrata* (Cham. & Schltdl.) Bocquet *subsp. tenella* (Tolmatchew) Bocquet	BOX4908	80289	27325	34,0	22869	83,7	22129	81,0
*Silene laxa* Boiss. & Kotschy	P451_114	1852	616	33,3	532	86,4	519	84,3
*Silene linneana* Voroschilov	BOX4911	59027	23255	39,4	20592	88,5	19920	85,7
*Silene muscipula* L.	P451_136	90084	26197	29,1	19196	73,3	18655	71,2
*Silene noctiflora* L.	P451_117	76525	22838	29,8	19390	84,9	18869	82,6
*Silene nutans* L.	P451_118	64147	26269	41,0	18865	71,8	18278	69,6
*Silene odontopetala* Fenzl	P451_119	102476	33481	32,7	20585	61,5	19914	59,5
*Silene sachalinensis* F. Schmidt	BOX5117	74045	26713	36,1	22083	82,7	21425	80,2
*Silene sedoides* Poir.	P451_122	78619	22727	28,9	18647	82,0	18144	79,8
*Silene soczaviana* Schischk.	BOX4885	72234	27429	38,0	21580	78,7	20861	76,1
*Silene vittata* Stapf	P451_123	65215	21172	32,5	18562	87,7	17943	84,7
*Viscaria vulgaris* Bernh.	P1844_106	73776	28644	38,8	20858	72,8	20201	70,5

## 4 Experimental Design, Materials and Methods

### 4.1 Sampling

The dataset contains 40 specimens from 34 taxonomic species, distributed across 17 sections of *Silene* and six closely related genera. Of the included species, 30 are diploid while three from section *Physolychnis* are polyploid. The set also include a putatively polyploid sample of *Agrostemma githago,* a species with several tetraploid (2n=48) chromosome counts. Effort was made to include species of various sections across the phylogenetic diversity of *Silene* (see [1]) in order to fill taxonomic gaps between previously sequenced transcriptomes. Samples used for RNA extraction were either grown from seeds in the botanical gardens in Gothenburg and Copenhagen or collected in the wild and preserved with RNAlater (Thermo Fisher Scientific, Inc., Wilmington, DE, USA). To maximize the number and coverage of sequenced transcripts we strived to include tissue from all stages of the plants life cycle i.e. roots, stem, leaves, developing buds, flowers, fruits. A complete list of included samples and their treatment before RNA extraction can be found in Table 1.

### 4.2 Laboratory procedures

“Oslo” dataset (Samples: BoxXXXX): Whole specimen plants were ground to a fine powder using mortar and pestle chilled on N_2_(l). RNA was extracted from 100 mg powder using the Spectrum^TM^ Plant Total RNA Kit (Sigma Aldrich, St. Louis, MO) according to manufacturer's instructions utilising “protocol A”, simultaneously employing on-column DNase I digestion. Mortars and pestles used for grinding frozen material were decontaminated between samples using 10% Sodium Hypochlorite. One microgram of total RNA was used as input for sequencing library construction using TruSeq^TM^ reagents (Illumina, San Diego, CA) according to manufacturer's instructions. Samples were indexed with 12 multiplex adapters and sequenced with v3 clustering and SBS reagents, employing 100 bp paired end reads on 2 lanes of an Illumina HiSeq 2000 at the Norwegian Sequencing Center in Oslo, Norway (http://www.sequencing.uio.no).

“Stockholm” dataset (Samples: P451_XXX / P1844_XXX): Plant tissue was ground in liquid nitrogen and stored at -80°C before RNA extraction. RNA was extracted from all samples following steps 2-7 from the protocol of Wang et. al. [2] and continued from step 2 in the RNAeasy plant kit (Qiagen, Inc., Valencia, CA, USA) manual. Quality and concentration of the extracted RNA was measured using NanoDrop (Thermo Fisher Scientific, Inc., Wilmington, DE, USA). RNA-seq library preparation and sequencing of the samples were performed at SciLifeLabs Facility in Stockholm, Sweden (https://www.scilifelab.se/). 2 × 100bp paired end read libraries were prepared using TruSeq Stranded Total RNA LT Sample Prep Kit - Set A (with Ribo-Zero Plant). (Serial number RS-122-2401.) using a custom protocol on an Agilent Bravo pipetting robot (Agilent, Santa Clara, USA). *Silene conoidea* and *Viscaria vulgaris* were sequenced using 2 × 125bp paired end reads on the Illumina HiSeq2500 at the SciLifeLabs Facility in Stockholm.

### 4.3 Data processing

Adapter removal and quality filtering of raw reads were performed using Trimmomatic [3] with settings “ILLUMINACLIP:TruSeq3-PE-2.fa:2:30:10 LEADING:3 TRAILING:3 SLIDINGWINDOW:4:20 MINLEN:36”, i.e. trimming trailing and leading bases with a quality score below 3, trimming reads when the average quality score drops below 20 in a 4 bp sliding window and keeping reads with a minimum length of 36. Quality control after trimming and filtering was performed using FastQC v.0.11.7 (http://www.bioinformatics.babraham.ac.uk/projects/fastqc). Filtered reads were assembled with Trinity v.2.9.1 [4] using default settings. Trinity works in a three-stage process by first performing a de-novo assembly of reads into contigs then proceeds with clustering the contigs and creating de Bruijn graphs for the complete sequences before adding the complete set of reads to the graphs. Finally, all paths within the graphs are traced to produce full length sequences for all transcripts, alternative splicing's and possible sequence variants. The sequences are then grouped into isogroups(Trinity “genes”), with alternative variants denoted as isoforms within each isogroup. Basic statistics on the number of isogroups, total number of isoforms, contig lengths and N50 values were acquired using the script TrinityStats.pl from the trinity package and parsed using a custom script. For each isogroup the longest isoform was extracted from the assembled contigs using the included scripts “get_longest_isoform_seq_per_trinity_gene.pl”.

### 4.4 Annotation

The assembled longest isoform contigs were searched against the Uniprot/Swiss-Prot database [5] using BLASTx v.2.2.31+ [6], using a e-value cutoff of E-4 and saving the top 5 hits for each query. For each top hit we downloaded information on connected GO-terms from the curated UniProt/SwissProt database. To exclude potential contaminants only hits with taxonomic identities within Magnoliopsida were included in the final set. Information on assigned GO-terms for each accession were downloaded from the UniProt/Swiss-Prot database.

## Limitations

None.

## Ethics Statement

The authors have read and follow the ethical requirements for publication in Data in Brief and confirm that the current work does not involve human subjects, animal experiments or any data collected from social media platforms.

## CRediT Author Statement

**Patrik Cangren:** Methodology, Data curation, Validation, Writing- Original Draft. **Yann Bertrand:** Methodology, Investigation, Conceptualization, **John Braverman:** Conceptualisation, Funding acquisition, **Gregor Duncan Gilfillan:** Investigation, **Matthew B. Hamilton:** Conceptualisation, Funding acquisition, **Bengt Oxelman:** Conceptualization, Supervision, Writing- Reviewing and Editing.

## Data Availability

Mendeley Data40 Transcriptomes from Sileneae (Original data).

## References

[1] Jafari F., Zarre S., Gholipour A., Eggens F., Rabeler R.K., Oxelman B. (2020). A new taxonomic backbone for the infrageneric classification of the species-rich genus Silene (Caryophyllaceae). Taxon.

[2] Wang G., Wang G., Zhang X., Wang F., Song R. (2012). Isolation of high quality RNA from cereal seeds containing high levels of starch. Phytochem. Anal..

[3] Bolger A.M., Lohse1 M., Usadel B. (2014). Trimmomatic: a flexible trimmer for illumina sequence data. Bioinformatics.

[4] Haas B.J., Papanicolaou A., Yassour M., Grabherr M., Blood P.D., Bowden J., Couger M.B., Eccles D., Li B., Lieber M., MacManes M.D., Ott M., Orvis J., Pochet N., Strozzi F., Weeks N., Westerman R., William T., Dewey C.N., Henschel R., LeDuc R.D., Friedman N., Regev A. (2013). De novo transcript sequence reconstruction from RNA-seq using the Trinity platform for reference generation and analysis. Nature Protocols.

[5] Boutet E., Lieberherr D., Tognolli M., Schneider M., Bansal P., Bridge A.J., Poux S., Bougueleret L., Xenarios I. (2016). UniProtKB/Swiss-Prot, the manually annotated section of the uniprot knowledgebase: how to use the entry view. Methods Mol. Biol..

[6] Camacho C., Coulouris G., Avagyan V., Ma N., Papadopoulos J., Bealer K., Madden T.L. (2009). BLAST+: architecture and applications. BMC Bioinformatics.

